# Supplementary material for: Costs of home-delivered antiretroviral therapy refills for persons living with HIV: Evidence from a pilot randomized controlled trial in KwaZulu-Natal, South Africa
Source: PLOS Glob Public Health. 2024 Dec 30;4(12):e0003368. doi: 10.1371/journal.pgph.0003368 (PMC11684705; doi:10.1371/journal.pgph.0003368)
Supplement: S1 File — (DOCX) [file pgph.0003368.s001.docx]

**S1 File. Narrative summary of the Deliver Health Study.**

Conducted from: October 2019 – December 2020

Compiled by: Ashley Tseng & Xolani Ntinga

**
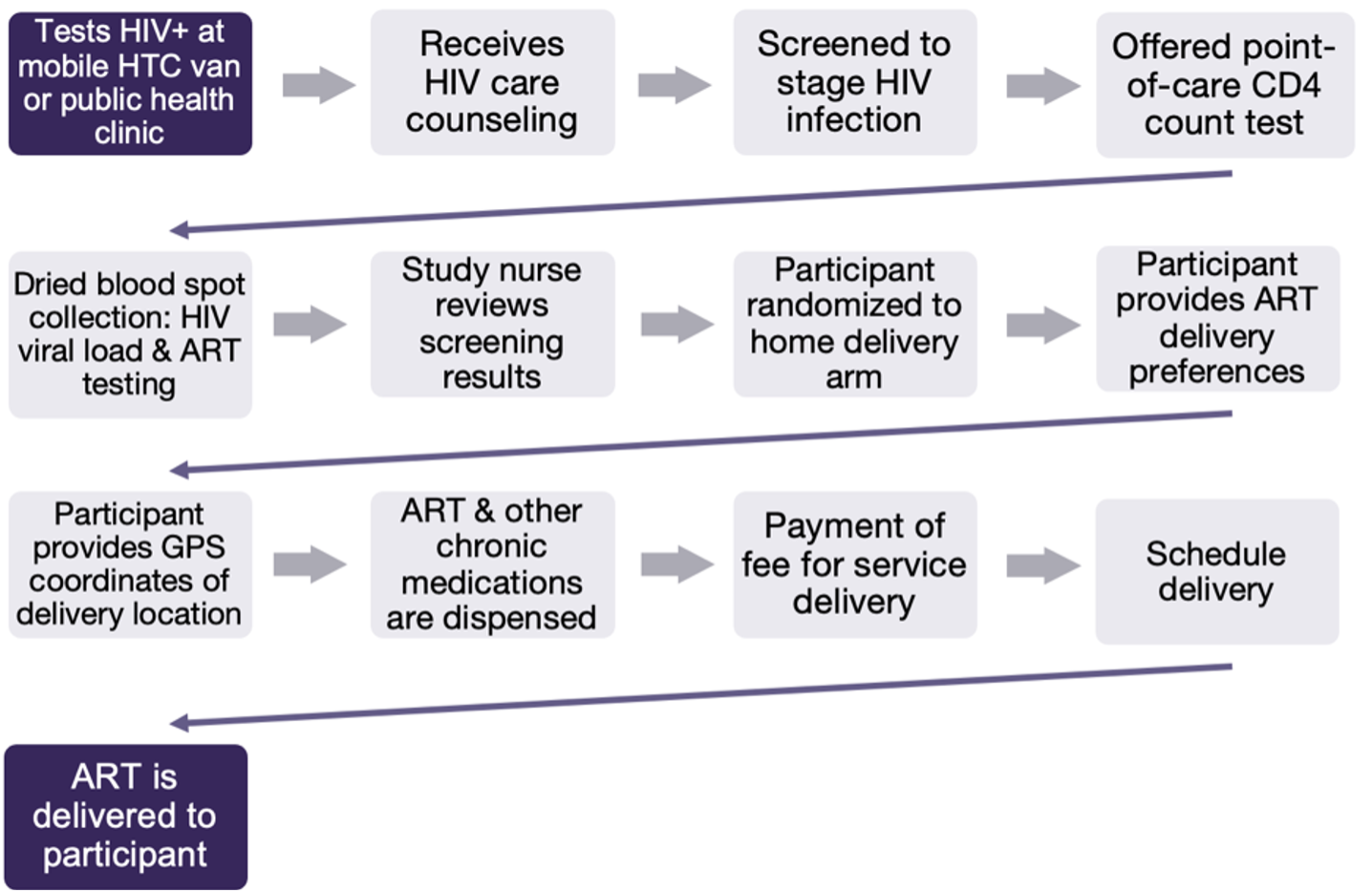
**

**Figure 1.1. Flow map of home-delivered ART resupply steps in the Deliver Health Study.**

**Table 1.1. Narrative description of Deliver Health Study antiretroviral therapy (ART) refill activities administered through the Human Sciences Research Council (HSRC) in Sweetwaters, KwaZulu-Natal, South Africa.**

| **Study activity** | **Deliver Health Study ART refill method** | |
| --- | --- | --- |
|  | **Home delivery** | **Clinic-based** |
| **Supply chain** | HSRC has a memorandum of understanding with the South African National Department of Health (NDoH) where they provide HSRC with HIV test kits and ART. The Deliver Health Study purchased all supplies for point-of-care tests including PIMA machines and PIMA cartridges (to measure CD4 cell counts) and blood collection tubes (to measure HIV viral load and determine viral suppression). The study had two dedicated HSRC fleet vehicles: a fleet bus (mobile clinic) and a Ford Ranger or similar vehicle (if only home delivery was needed). | The South Africa NDoH provided all public health facilities with ART and other medications, and all the resources the clinics used (e.g., computers, supplies). |
| **Community sensitization and study recruitment** | The Deliver Health Study worked with local HIV clinics to establish procedures for registration of ART clients, ART supply procurement, and clinic referrals when needed for the community-based participants.^a^  Recruitment at HIV clinics: Outreach for recruitment was conducted by study staff at five public health clinics to identify people living with HIV and who may be engaged in care. If individuals were interested in participating in the study, then they were given the Deliver Health Study phone number to send a “please call me” request. Upon receiving the request, a study staff member would call the individual to provide more information about the study.  Recruitment at community-based venues: The Deliver Health Study team conducted community mobilization talks with groups of community members to encourage HIV testing through discussions around HIV and HIV testing at taxi ranks, taverns, and other community hotspots. | |
| **Training** | All study staff were trained in all study activities. Over the course of one work week, the Deliver Health Study data manager and research coordinator administered trainings at HSRC on data collection (e.g., how to use REDCap, how to complete case report forms in REDCap, how to set up REDCap on a mobile device, how to use the GPS devices for home delivery) and on the following study activities:   - Home HIV testing and counseling - Mobile HIV testing and counseling - CD4 testing - Dried blood spot card collection - Blood tube collection - Interpretation of screening tests   In implementation, the study nurse primarily focused on clinical activities. | |
| **Enrollment** | Participants who were HIV-positive and ART-eligible according to national guidelines (2023 ART Clinical Guidelines for the Management of HIV in Adults, Pregnancy and Breastfeeding, Adolescents, Children, Infants and Neonates) were eligible to continue with enrollment. Each potential study participant provided written informed consent to the study. Participants were provided with a unique identifier to enable tracking of participants through each stage of the continuum of care (testing, visiting a clinic, initiating ART, monitoring, and collecting ART refills). | |
| **Randomization** | Randomization was stratified by community or clinic and blocked to ensure balance. The randomization sequence was designed at the University of Washington International Clinical Research Center and programmed into the mobile phone for each participant/household enrolled. The randomization arm was not determined until the participant had completed all screening procedures.  For participants who were already engaged in HIV care at a public health clinic, study staff informed the clinic that the participant was not lost to follow-up but would be working with HSRC for the Deliver Health Study.  Participants in the fee for delivery arm received tailored ART delivery to their location of choice (home, work, etc.). At the randomization visit, the following procedures took place:   - Participant completed the detailed delivery preferences questionnaire - Staff collected GPS coordinates of the delivery location - Nurse dispensed ART and other chronic medications - Staff collected payment of the fee for service delivery* - Scheduled delivery for 1 month follow-up visit   *Nominal fee on a tiered system based on socio-economic status (ZAR 30-90 = USD 2-7). An invoice was provided to each participant. Cash received was logged and stored in a secure lock box. If delivery fees were not paid by month five, then a 20-day notice was given and the participant was transferred back to ART collection at the clinic. | Randomization was stratified by community or clinic and blocked to ensure balance. The randomization sequence was designed at the University of Washington International Clinical Research Center and programmed into the mobile phone for each participant/household enrolled. The randomization arm was not determined until the participant had completed all screening procedures.  At randomization, for participants who were not currently taking ART, the study nurse followed local guidelines on ‘test and treat’ for HIV and supplied ART and a referral to the clinic. If the participant was currently taking ART, the study nurse documented the clinic the participant was receiving care from. |
| **ART dispensation** | Study nurses dispensed ART (TDF/FTC/EFV: Tenofovir/Emtricitabine/Efavirenz [Atripla/FDC]) and any other chronic medications the participants were taking. For participants who were already engaged in HIV care at baseline, the study nurse went to the clinics to verify active participant engagement in care and check what other medications they were on, then dispensed all medications at HSRC and loaded onto the home delivery vehicle. | The pharmacist at each public health clinic dispensed ART and any other medications. |
| **ART refill visits at months 1, 3, 6, and exit** | Participants in the home delivery arm had visits at 1 month and 3 months after enrollment to deliver their medication. At each follow-up visit, the following procedures took place:   - Completed health questionnaire - Dispensed medication (ART and other chronic medications) - Blood draw for HIV viral load testing - Took dried blood spots - Measured CD4 counts with PIMA cartridges and PIMA machine - Scheduled following visit   The Deliver Health Study home delivery team comprised of 3 staff members: 1 nurse, 1 driver/data collector, and 1 data collector. Later in the study (during the first COVID-19 wave in South Africa), the home delivery team was reduced to a team of 1 due to COVID-19 restrictions – the nurse also became a driver and data collector.^b^ The team made home deliveries three days per week. | Participants received an optimized ART linkage package, including a clinic referral card to support linkage to ART.  At months 1 and 3 visits, participants randomized to the clinic arm received a phone call to record if the participant had visited the clinic, initiated ART (if not already on ART at enrollment), and if they had picked up their refills while at the clinic. |
| **Chart abstractions** | The study team reviewed participant medical records at clinics to record any visits to the clinic and the reason. | Clinic chart abstractions included the following information: patient name, date of birth, next ART pickup dates, what medication(s) they were on. |
| **Study exit** | At the in-person exit visit, either at a community location in the mobile van or at the participant’s home, the following procedures took place:   - Completed health questionnaire (uptake of HIV care, clinic visits, ART initiation, ART adherence, barriers to care) - Completed acceptability questionnaire on home delivery - Dispensed medication (ART and other chronic medications) - Blood draw for HIV viral load testing (up to 6 mL), the results of which was provided to participants to support their HIV care - Referral to a public health clinic   - For people living with HIV who were *newly diagnosed* in the study: after the study concluded, study staff referred these individuals to a public health clinic and notified the clinics of new diagnoses, providing the selected clinic with study clinical records   - For people living with HIV who *already knew their HIV status* at baseline: these individuals “exited” their clinic care during participation in the study and resumed standard clinic care after the study ended; study staff provided clinics with study clinical records | At the in-person exit visit, either at a community location in the study mobile van or at the participant’s home, the following procedures took place:   - Health questionnaire (uptake of HIV care, clinic visits, ART initiation, ART adherence, barriers to care) - Blood draw for HIV viral load testing (up to 6 mL), the results of which was provided to participants to support their HIV care |

Notes:

^a^The Deliver Health Study data collectors and study nurse recruited participants in the Deliver Health Study. There were no community outreach workers working on participant recruitment for the study outside of the data collectors and study nurse who were already hired with the Deliver Health Study team. For programmatic intervention, since there would be no data collectors (a research-specific role), we assumed there would be one community outreach worker who would be recruiting people living with HIV for the home-delivered ART service.

^b^For programmatic implementation, we assumed there would be a home-delivery team of 2 individuals: 1 driver and 1 nurse.
