# Supplementary material for: Costs of home-delivered antiretroviral therapy refills for persons living with HIV: Evidence from a pilot randomized controlled trial in KwaZulu-Natal, South Africa
Source: PLOS Glob Public Health. 2024 Dec 30;4(12):e0003368. doi: 10.1371/journal.pgph.0003368 (PMC11684705; doi:10.1371/journal.pgph.0003368)
Supplement: S2 File — (DOCX) [file pgph.0003368.s002.docx]

**S2 File: Additional methods.**

Table of Contents

[2.1. Detailed methods 2](#_Toc177826313)

[2.1.1. Optimized home delivery algorithm 2](#_Toc177826314)

[2.1.2. Detailed methods for costing of clinic-based ART refills and care 2](#_Toc177826315)

[2.1.3. Detailed description of outcomes 2](#_Toc177826316)

[2.2. Costing categories and sources 4](#_Toc177826317)

[2.3. As-observed and programmatic scenario assumptions 11](#_Toc177826318)

[2.3.1. Duration of ART initiation and ART refill visits based on time-and-motion study 11](#_Toc177826319)

[2.3.2. As-observed scenario summary 11](#_Toc177826320)

[2.3.3. Programmatic scenario of home-delivery intervention assumptions 11](#_Toc177826321)

[2.4. Converted cost estimates from the literature to 2022 USD 13](#_Toc177826322)

[References 16](#_Toc177826323)

# 2.1. Detailed methods

## 2.1.1. Optimized home delivery algorithm

Clients randomized to the home-delivered antiretroviral therapy (ART) and monitoring arm provided their delivery preferences and paid a one-time fee for the home delivery service at the baseline visit (or a following visit if the client was unable to pay at baseline). A novel feature of the Deliver Health Study was that an algorithm was developed in collaboration with Amazon data scientists to generate optimized driving routes for the home delivery of ART [1]. The algorithm solved the “travelling salesman problem” [2] to optimize the weekly deliveries and considered the following parameters: participants who will run out of ART in the next month, participant locator information (address or geographic coordinates of their pre-specified delivery location, usually the home), and participant availability. Delivery preferences were based on pre-specified days and time windows during which the study team planned for home deliveries, specifically on Tuesdays and Thursdays between 10:00 AM and 7:00 PM and Saturdays between 8:00 AM and 4:00 PM. The nominal service fee for home delivery was determined through community engagement and based on a tiered system proportional to the client’s income level, self-reported at study enrollment. If delivery fees were not paid by month 5, then a 20 days’ notice was given, and the client was transferred to ART collection at a public health clinic. All participants received follow-up calls and reminders from the study staff.

## 2.1.2. Detailed methods for costing of clinic-based ART refills and care

To estimate costs for clinic-based care, we obtained actual spending values for KwaZulu-Natal province for financial years 2019 and 2020 from the South African National Treasury [3]. In South Africa, the financial year period occurs from April 1 to March 31 in the following year. In order to mirror the 15-month period of implementation in the Deliver Health Study for a more accurate cost comparison, we estimated clinic financial year 2019 costs for the 6-month period of October 1, 2019-March 31, 2020 and financial year 2020 costs for the 9-month period of April 1-December 31, 2020. We multiplied the 15-month total estimate by 0.8 to scale to a 12-month (annual) estimate.

For training, vehicles, equipment, building, utilities, personnel, drugs, laboratory and clinical supplies, and miscellaneous costs, we used the ﻿2020 HIV/AIDS Component of the HIV, TB, Malaria and Community Outreach Grant report [4] to obtain the proportion of comprehensive HIV/AIDS program that was spent on ART-related care and applied that proportion to the value of each costing category. Spending on ART care in KwaZulu-Natal for 2019-2020 was estimated to be ZAR 4,206,397 and spending on the comprehensive HIV/AIDS program in KwaZulu-Natal for 2019-20 was approximately ZAR 5,206,664, thus the proportion of ART care of the comprehensive HIV/AIDS program in KwaZulu-Natal for 2019-2020 was $\mathrm{ZAR} \frac{4,206,397}{5,206,664}=0.8079$.

## 2.1.3. Detailed description of outcomes

In the Deliver Health Study, when a client was first diagnosed with HIV and initiated in care, they were prescribed a one-month supply of ART and had a follow-up visit one month after their initiation visit. At the month 1 follow-up visit, they received HIV care counseling and were prescribed a two-month supply of ART, and had their next follow-up visit two months later. At the month 3 follow-up visit, they were prescribed a 3-month supply of ART and had follow-up refill visits every 3 months thereafter. Thus, the first-year costs of home delivery comprised the initiation visit, month 1, month 3, month 6, and month 9 refill visits. The cost of home-delivered ART refills and monitoring in subsequent years were estimated based on ART refill visits every 3 months for a total of 4 follow-up visits per year. Costs in the first year (start-up costs) and subsequent years were reported for the home delivery intervention, while annual costs were reported for standard clinic-based care since we did not have data on the start-up costs from clinics. The total sum of fees paid by clients for the home delivery service were subtracted from the total programmatic cost.

# 2.2. Costing categories and sources

**Supplementary Table 2.2.1.** **Home-delivered ART refills and monitoring costing categories and sources.**

| Type of cost | Cost category name | Data year | Data source | Notes |
| --- | --- | --- | --- | --- |
| Fixed costs | Hiring | 2017 | DO ART Study [5] | Discount rate of 3% - assumed hiring was a process that needs to be repeated every few years. |
|  | Training | 2017 | DO ART Study [5] | Discount rate of 3% - assumed training was a process that needs to be repeated every few years. |
|  | Community outreach/  mobilization | 2017 | DO ART Study [5] |  |
|  | Vehicles | 2014, 2015, and 2017 | DO ART Study [5] | Vehicle costs: There was one dedicated Ford Ranger and one fleet bus (mobile clinic) vehicle for use by the study team, but only one vehicle was used at a given time since there was only one team (of 3 study staff) in the field making home deliveries. Therefore, each vehicle’s costs were divided by 2 to account for being used roughly half the time.   - Ford Ranger purchased June 15, 2015: Annuity factor calculated with a discount rate of 3% and 10 years of useful life. Total cost divided by 2 to account for use 50% of the time in study. - Human Sciences Research Council fleet bus (mobile clinic) purchased June 15, 2014: annuity factor calculated with a discount rate of 3% and 10 years of useful life. Total cost divided by 2 to account for use 50% of the time in study.   Vehicle maintenance costs in 2017: Annuity factor calculated with a discount rate of 3% and 1 year of useful life. |
|  | Equipment | 2016,  2019 | DO ART Study [5]; Deliver Health Study [1] | Discount rate of 3%. Assumed the same equipment costs for Deliver Health Study as the DO ART Study.  Specific costs from the Deliver Health Study:   - Smartphones (Android) - Laptops - GPS trackers |
|  | Building | 2016 | Human Sciences Research Council (obtained in DO ART Study [5]) | Assumed the same building (HSRC) costs for Deliver Health Study as the DO ART Study. Office rental was included at 100% and central office at 10%. |
|  | Utilities | 2015 | Human Sciences Research Council (obtained in DO ART Study [5]) | Data usage was included in this category at 100%; all other cost items are included at 10%. |
|  | Personnel | 2017, 2019, 2020 | *See notes* | As implemented in Deliver Health Study [1]:   - Salaries from Deliver Health Study budget (2019) [1]: Primary Investigator, Study Implementation, Study Coordinator, Driver, Research Assistant, Data Collector - All other salaries from DO ART Study (2017) [5]: Study Nurse, Community Outreach Worker, Data Specialist, Study Logistics, Human Resources, Accountant   As implemented by the South African National Department of Health:   - Professional Nurse Grade 2 (General Nursing) salary (notch 4, full-time) from FY2019 SA DPSA salary data [6]: ZAR 342,033 - All other salaries from DO ART Study (2017) [5]: Senior Program Manager, Team Leader, Community Outreach Worker, Human Resources, Accountant, Driver, Data Specialist   *Note: for the 6-month and 12-month refill scenarios, the annual values were divided by 2 and 4, respectively, to account for a reduction in visits from 4 visits per year (for the standard 3-month refill scenario) to 2 visits and 1 visit per year, respectively. |
| Variable costs | Drugs | 2024 | South African National Department of Health Master Health Product List January 2024 [7] | All participants in the Deliver Health Study were on the TDF/FTC/EFV (Tenofovir/Emtricitabine/Efavirenz or Atripla/FDC) ART regimen which is one tablet taken once orally daily [8]  The cost of ART drugs is negotiated through a tender process with the South African National Department of Health and are fixed for a number of years, thus we did not adjust the ART costs for inflation [9]. This was the only item for which did not adjust for inflation.  *Note: for the 6-month and 12-month refill scenarios, the annual values were divided by 2 and 4, respectively, to account for a reduction in visits from 4 visits per year (for the standard 3-month refill scenario) to 2 visits and 1 visit per year, respectively. |
|  | Labs | 2016 | DO ART Study [5] | Assumed the same lab consumables costs for Deliver Health Study as the DO ART Study.  *Note: for the 6-month and 12-month refill scenarios, the annual values were divided by 2 and 4, respectively, to account for a reduction in visits from 4 visits per year (for the standard 3-month refill scenario) to 2 visits and 1 visit per year, respectively. |
|  | Materials and clinical supplies | 2016 | DO ART Study [5] | Assumed the same clinical supplies costs for Deliver Health Study as the DO ART Study.  *Note: for the 6-month and 12-month refill scenarios, the annual values were divided by 2 and 4, respectively, to account for a reduction in visits from 4 visits per year (for the standard 3-month refill scenario) to 2 visits and 1 visit per year, respectively. |
|  | Fuel | 2019 | *See notes* | - Distance calculations using data from the Deliver Health Study [1] - Diesel prices from November 2019 [10] - Estimated fuel efficiency of Ford Ranger (km/L) [11]   *Note: for the 6-month and 12-month refill scenarios, the annual values were divided by 2 and 4, respectively, to account for a reduction in visits from 4 visits per year (for the standard 3-month refill scenario) to 2 visits and 1 visit per year, respectively. |
|  | Administrative overhead | 2019 | Deliver Health Study [1] | 8% of all costs except for ART. |
| Fees | Fee for home delivery service | 2019 | Deliver Health Study [1] | Clients in the home delivery arm paid a one-time fee for the home delivery service at the baseline/randomization visit. The fee was determined based on a sliding scale proportional to the client’s income (self-reported at study enrollment). The total sum of fees paid by clients and subtract that from the total programmatic cost (before dividing by the # of clients and # of virally suppressed clients). |

**Cost estimation steps for home-delivered ART refills and monitoring:**

1. For all costs reported in ZAR (except for ART drugs), they were first inflation-adjusted using the World Bank GDP implicit deflator for South Africa [12] from the year the data were collected to 2022 ZAR, then converted to 2022 USD based on the average ZAR to USD exchange rate in 2022 [13]. For costs reported in USD, they were inflation-adjusted to 2022 USD using the United States annual average Consumer Price Index [14].
2. Calculating average annual costs:
   1. Cost per client: We divided the total cost by the number of participants in the home-delivery group of the Deliver Health Study with successful follow-up (81 participants)
      1. Note: The total sum of fees paid by clients in the home-delivery group of the Deliver Health Study (ZAR 4686 or US $286) was deducted from the sum of fixed and variable costs to obtain the total cost
   2. Cost per client virally suppressed: We divided the total cost by the number of participants in the home-delivery group of the Deliver Health Study with successful follow-up and who were virally suppressed at study exit (71 participants)
      1. Note: The total sum of fees paid by clients in the home-delivery group of the Deliver Health Study (ZAR 4686 or US $286) was deducted from the sum of fixed and variable costs to obtain the total cost

**Supplementary Table 2.2.2.** **Clinic-based ART refills and care costing categories and sources.**

| Type of cost | Cost category name | Data year | Data source | Notes |
| --- | --- | --- | --- | --- |
| Fixed costs | Hiring |  |  | Assuming that hiring costs were included in “Training and development” |
|  | Training | April 1, 2019-March 31, 2020 | South African Department of Treasury (Vulekamali)^a^ [3] | Includes:   - Training and development |
|  | Community outreach/  mobilization | April 1, 2019-March 31, 2020 | South African Department of Treasury (Vulekamali)^a^ [3] | Includes:   - Advertising - Communication (G&S) |
|  | Vehicles | April 1, 2019-March 31, 2020 | South African Department of Treasury (Vulekamali)^a^ [3] | Includes:   - Fleet services (including government motor transport) - Rental and hiring - Transport provided: Departmental activity |
|  | Equipment | April 1, 2019-March 31, 2020 | South African Department of Treasury (Vulekamali)^a^ [3] | Includes:   - Other machinery and equipment - Minor assets - *Transport equipment (ZAR 0 for 2019/2020)* |
|  | Building | April 1, 2019-March 31, 2020 | South African Department of Treasury (Vulekamali)^a^ [3] | Includes:   - Operating leases - Property payments - *Venues and facilities (ZAR 0 for 2019/2020)* |
|  | Utilities | April 1, 2019-March 31, 2020 | South African Department of Treasury (Vulekamali)^a^ [3] | Includes:   - Operating payments |
|  | Personnel | April 1, 2019-March 31, 2020 | South African Department of Treasury (Vulekamali)^a^ [3] | Includes:   - Salaries and wages - Social contributions - Agency and support / outsourced services - Contractors - *Consultants and professional services: Business and advisory services (ZAR 0 for 2019/2020)* |
| Variable costs | Drugs | 2024 | South African National Department of Health Master Health Product List January 2024 [7] | We assumed the only ART regimen being used was TDF/FTC/EFV to properly estimate costs relative to the Deliver Health Study (all participants in the Deliver Health Study were on the TDF/FTC/EFV regimen).  We had the cost of TDF/FTC/EFV from the South African National Department of Health Master Product List from January 2024, which was ZAR 287.57 for 84 tablets. The cost of ART drugs is negotiated through a tender process with the South African National Department of Health and are fixed for a number of years, thus we did not adjust the ART costs for inflation (Meyer-Rath et al., 2019). This was the only item for which did not adjust for inflation. All other costs were inflated to 2022 ZAR, then converted from 2022 ZAR to 2022 USD.  Notes: The spreadsheet downloaded from Vulekamali included an “Inventory: Medicine” expense category under the HIV/AIDS program, but we opted to use the cost of TDF/FTC/EFV from the NDOH because the “Inventory: Medicine” category could have included other HIV-related non-ART medicines (e.g., PrEP) which would overestimate the cost of ART intervention only. For the 6-month and 12-month refill scenarios, the annual values were divided by 2 and 4, respectively, to account for a reduction in visits from 4 visits per year (for the standard 3-month refill scenario) to 2 visits and 1 visit per year, respectively. |
|  | Labs | April 1, 2019-March 31, 2020 | South African Department of Treasury (Vulekamali)^a^ [3] | Includes:   - Laboratory services   *Note: for the 6-month and 12-month refill scenarios, the annual values were divided by 2 and 4, respectively, to account for a reduction in visits from 4 visits per year (for the standard 3-month refill scenario) to 2 visits and 1 visit per year, respectively. |
|  | Materials and clinical supplies | April 1, 2019-March 31, 2020 | South African Department of Treasury (Vulekamali)^a^ [3] | Includes:   - Consumable supplies - Consumable: Stationery, printing and office supplies - Inventory: Clothing material and accessories - Inventory: Materials and supplies - Inventory: Medical supplies - Inventory: Other supplies   *Note: for the 6-month and 12-month refill scenarios, the annual values were divided by 2 and 4, respectively, to account for a reduction in visits from 4 visits per year (for the standard 3-month refill scenario) to 2 visits and 1 visit per year, respectively. |
|  | Administrative overhead | April 1, 2019-March 31, 2020 | South African Department of Treasury (Vulekamali)^a^ [3] | Includes:   - Administrative fees   *Note: for the 6-month and 12-month refill scenarios, the annual values were divided by 2 and 4, respectively, to account for a reduction in visits from 4 visits per year (for the standard 3-month refill scenario) to 2 visits and 1 visit per year, respectively. |
|  | Miscellaneous | April 1, 2019-March 31, 2020 | South African Department of Treasury (Vulekamali)^a^ [3] | Includes:   - Catering: Departmental activities - Travel and subsistence   *Note: for the 6-month and 12-month refill scenarios, the annual values were divided by 2 and 4, respectively, to account for a reduction in visits from 4 visits per year (for the standard 3-month refill scenario) to 2 visits and 1 visit per year, respectively. |
|  | Fuel |  |  | Assuming that fuel costs were included in the “Vehicles” category |

^a^Extracted value for HIV/AIDS program in KwaZulu-Natal for 2019 and 2020 from the [“Budgeted and Actual Provincial Expenditure” spreadsheet](https://uwnetid-my.sharepoint.com/:x:/r/personal/atseng23_uw_edu/Documents/UW%20PhD/Dissertation/Dissertation%20Research/Aim%203_Cost%20Impact/Costing/Deliver%20Health%20Study/Costing%20Data%20Sources/SA%20National%20Treasury/Vulekamali/Budgeted%20and%20Actual%20Estimates%20of%20Provincial%20Expenditure%20of%20South%20Africa%202023-24.xlsx?d=w4e9d4d2fad254de8b51f5f47fc2867fb&csf=1&web=1&e=jFgbcy) downloaded from [Vulekamali](https://vulekamali.gov.za/datasets/budgeted-and-actual-provincial-expenditure/budgeted-and-actual-provincial-expenditure). The period of coverage for the 2019 financial year is April 1, 2019-March 31, 2020. The period of coverage for the 2020 financial year is April 1, 2020-March 31, 2021. Vulekamali pulls data from the Estimates of Provincial Revenue and Expenditure (EPRE) tables published on the South African [National Treasury website](https://www.treasury.gov.za/documents/provincial%20budget/default.aspx).

**Cost estimation steps for clinic-based ART refills and care:**

1. For training, vehicles, equipment, building, utilities, personnel, drugs, labs, clinical supplies, and miscellaneous costs, we used the 2020 HIV/AIDS programme spending report [4] to obtain proportion of comprehensive HIV/AIDS program that was spent on ART-related care, and applied that proportion to the value of each costing category:
   1. Spending on ART care in KZN for 2019-20: ZAR 4,206,397
   2. Spending on the comprehensive HIV/AIDS program in KZN for 2019-20: ZAR 5,206,664

🡪 Proportion of ART care of the comprehensive HIV/AIDS program in KZN for 2019-20: $\mathrm{ZAR} \frac{4,206,397}{5,206,664}=0.8079$

1. For all costs except for ART drugs, they were first inflation-adjusted using the World Bank GDP implicit deflator for South Africa [12] from the year the data were collected to 2022 ZAR, then converted to 2022 USD based on the average ZAR to USD exchange rate in 2022 [13]
2. Calculating average annual costs:
   1. Cost per client: We divided the total cost by the number of adults (aged 15 years and older) living with HIV in KwaZulu-Natal
      1. Total number of adults (aged 15 years and older) living with HIV in KwaZulu-Natal in September 2022 [15]: 1,925,698 people
   2. Cost per client virally suppressed: We divided the total cost by the number of adults (aged 15 years and older) living with HIV with suppressed viral load in KwaZulu-Natal
      1. We multiplied the estimated proportion of adults (15+) living with HIV with suppressed viral load in South Africa in 2020 (0.66) [16] by the total number of adults (15+) living with HIV in KwaZulu-Natal (1,925,698 people) [15] to obtain the number of adults (15+) living HIV in KwaZulu-Natal estimated to be virally suppressed in 2022: 1,270,961 people

# 2.3. As-observed and programmatic scenario assumptions

## 2.3.1. Duration of ART initiation and ART refill visits based on time-and-motion study

As a follow-up to the pilot Deliver Health Study, the SMART ART Study is an ongoing clinical trial in the same study area trialing home delivery of ART and community-based pick-up of ART, in comparison to standard clinic-based refills [17]. A time-and-motion study was conducted in January–February 2023 as part of the SMART ART Study at four public health clinics in the study area and of home delivery refill visits. For our study, we used the SMART ART Study time-and-motion results to inform the duration of ART initiation vs. refill visits. In the time-and-motion study, there were only two home delivery visits observed and both were ART resupply visits, thus we did not have an initiation visit to estimate staff time for. There were 14 clinic observations, 13 of which were ART resupply visits and one was an initiation visit. In terms of total staff time, the one initiation visit took 1 hour 19 minutes, compared to an average of 24 minutes and 42 seconds across the 13 refill visits at clinics. Therefore, we assumed an ART initiation visit (which includes HIV counselling) would take 3 times as long as an ART refill visit.

## 2.3.2. As-observed scenario summary

In the Deliver Health Study, the team made home deliveries 3 days per week and the average time spent driving per delivery trip was 91.07 minutes. The start/end location was the Human Sciences Research Council (HSRC) office in Sweetwaters, KwaZulu-Natal, South Africa. The Deliver Health Study home-delivery team comprised of 3 staff members: 1 nurse, 1 driver/data collector, and 1 data collector. The data collectors and study nurse recruited participants in the Deliver Health Study. There were no community outreach workers working on participant recruitment for the study outside of the data collectors and study nurse who were already hired with the Deliver Health Study team. Later in the study (during the first COVID-19 wave in South Africa), the home-delivery team was reduced to a team of 1 due to COVID-19 restrictions – the nurse also became a driver and data collector. The team made home deliveries 3 days per week.

## 2.3.3. Programmatic scenario of home-delivery intervention assumptions

In the programmatic scenario of the home-delivery intervention, we assumed the following:

- No data collector in programmatic implementation (since it is a research-specific role)
- Dedicated community outreach worker to recruit people living with HIV
- Home-delivery team of 2 individuals: 1 nurse and 1 driver
- 40-hour work weeks (8 hours per day, 5 days per week)
  - 6 hours for home deliveries
  - 1 hour to prepare/load delivery vehicle
  - 1 hour to unload delivery vehicle after

**Supplementary Table 2.3.1.** **Percent of staff time spent on the home delivery of antiretroviral therapy intervention in the Deliver Health Study.**

| Deliver Health Study position title | Equivalent position title based on South Africa National Department of Health salaries | Number of staff | % of total working hours spent on home delivery intervention | Assumptions |
| --- | --- | --- | --- | --- |
| Study Nurse | Professional Nurse (Grade 2, General Nursing, notch 4) | 1 | 27% | Calculation: $(91.07 minutes/60)*3 days per week) + ((1 hour pre-home deliveries + 1 hour post-home deliveries)*3 days per week)) /(40 hours per week) = 27\%$ |
| Driver | Driver | 1 | 27% | Calculation: $(91.07 minutes/60)*3 days per week) + ((1 hour pre-home deliveries + 1 hour post-home deliveries)*3 days per week)) /(40 hours per week) = 27\%$ |
| Community Outreach Worker | Community Outreach Worker | 1 | 25% | Works for HSRC to help with participant recruitment into the Deliver Health Study. Assumed 25% of their total working hours were spent on participant recruitment for the Deliver Health Study. |
| Study Coordinator | Team Leader | 1 | 25% | Assumed 25% of total working hours on the Deliver Health Study were spent directly on managing the home deliveries. |
| Human Resources | Human Resources | 1 | 5% | Works for HSRC to help with hiring of study staff (e.g., advertising position, compiling paperwork, etc.) |
| Accountant | Accountant | 1 | 5% | Works for HSRC to help with ordering study supplies, manage budgets, etc. |

# 2.4. Converted cost estimates from the literature to 2022 USD

Cost estimates from the literature were inflation-adjusted to 2022 USD using the United States annual average Consumer Price Index [14] for comparability to our study estimates.

**Supplementary Table 2.4.1.** **Barnabas et al. 2020 DO ART Study estimates of South Africa. [5]**

| **Cost estimate** | **Original Cost (reported in 2018 USD)** | **GDP Inflator from 2018 to 2022 USD** | **Total Cost in 2022 USD** |
| --- | --- | --- | --- |
| Midlands KZN annual cost per client (first year), community-based ART, steady-state (programmatic) scenario | 523.00 | 1.17 | 610 |
| Midlands KZN annual cost per client (subsequent years), community-based ART, steady-state (programmatic) scenario | 427.00 | 1.17 | 498 |
| Midlands KZN annual cost per client virally suppressed (first year), community-based ART, steady-state (programmatic) scenario | 759.00 | 1.17 | 885 |
| Midlands KZN annual cost per client virally suppressed (subsequent years), community-based ART, steady-state (programmatic) scenario | 678.00 | 1.17 | 790 |
| Midlands KZN annual cost per client (subsequent years), facility-based ART, efficient at-scale scenario | 249.00 | 1.17 | 290 |
| Midlands KZN annual cost per client virally suppressed (subsequent years), facility-based ART, efficient at-scale scenario | 422.00 | 1.17 | 492 |
| Midlands KZN annual cost per client (first year), community-based ART, efficient at-scale scenario | 312.00 | 1.17 | 364 |
| Midlands KZN annual cost per client (subsequent years), community-based ART, efficient at-scale scenario | 246.00 | 1.17 | 287 |
| Midlands KZN annual cost per client virally suppressed (first year), community-based ART, efficient at-scale scenario | 452.00 | 1.17 | 527 |
| Midlands KZN annual cost per client virally suppressed (subsequent years), community-based ART, efficient at-scale scenario | 390.00 | 1.17 | 455 |

**Supplementary Table 2.4.2. Meyer-Rath et al. 2019 estimates of South Africa. [9]**

| **Annual cost per client** | **Original Cost (reported in 2018 USD)** | **GDP Inflator from 2018 to 2022 USD** | **Total Cost in 2022 USD** |
| --- | --- | --- | --- |
| Midlands KZN (first year) | 249.00 | 1.17 | 290 |

**Supplementary Table 2.4.3. Guthrie et al. 2022 estimates of Uganda. [18]**

| **Cost estimate** | **Original Cost (reported in 2018 USD)** | **GDP Inflator from 2018 to 2022 USD** | **Total Cost in 2022 USD** |
| --- | --- | --- | --- |
| SOC Facility-based individual management (FBIM) annual cost per client (first year) | 157.93 | 1.17 | 184 |
| SOC Facility-based individual management (FBIM) annual cost per client (second year) | 152.49 | 1.17 | 178 |
| Community drug distribution points (CDDP) annual cost per client virally suppressed (first year) | 166.85 | 1.17 | 194 |
| Community drug distribution points (CDDP) annual cost per client virally suppressed (second year) | 146.42 | 1.17 | 171 |

**Supplementary Table 2.4.4. Prust et al. 2017 estimates of Malawi. [19]**

| **Total ART costs per patient** | **Original Cost (reported in 2016 USD)** | **GDP Inflator from 2016 to 2022 USD** | **Total Cost in 2022 USD** |
| --- | --- | --- | --- |
| Facility-based multi-month scripting (MMS): 3-month scripts | 121.41 | 1.22 | 148 |

**Supplementary Table 2.4.5. Benade et al. 2023 estimates of Zimbabwe. [20]**

| **Total average annual cost of HIV treatment per patient** | **Original Cost (reported in 2020 USD)** | **GDP Inflator from 2020 to 2022 USD** | **Total Cost in 2022 USD** |
| --- | --- | --- | --- |
| Community ART refill groups with 3-month dispensing | 178.00 | 1.13 | 201 |
| Community ART refill groups with 6-month dispensing | 167.00 | 1.13 | 189 |

**Supplementary Table 2.4.6. Nichols et al. 2021 estimates of Lesotho. [21]**

| **Average total annual cost of providing HIV care and treatment per patient** | **Original Cost (reported in 2018 USD)** | **GDP Inflator from 2018 to 2022 USD** | **Total Cost in 2022 USD** |
| --- | --- | --- | --- |
| 3-month facility-based arm | 122.28 | 1.17 | 143 |
| 3-month community adherence groups | 114.20 | 1.17 | 133 |
| 6-month community ART distribution | 112.58 | 1.17 | 131 |

# References

1. Barnabas R V, Szpiro AA, Ntinga X, Mugambi ML, van Rooyen H, Bruce A, et al. Fee for home delivery and monitoring of antiretroviral therapy for HIV infection compared with standard clinic-based services in South Africa: a randomised controlled trial. Lancet HIV. 2022;9: e848–e856. doi:10.1016/S2352-3018(22)00254-5

2. Letchford AN, Lodi A. Mathematical Programming Approaches to the Traveling Salesman Problem. Wiley Encyclopedia of Operations Research and Management Science. Wiley; 2011. doi:10.1002/9780470400531.eorms0505

3. South African National Treasury. Budgeted and Actual Provincial Expenditure. In: Vulekamali [Internet]. 2024 [cited 29 Jan 2024]. Available: https://vulekamali.gov.za/datasets/budgeted-and-actual-provincial-expenditure/budgeted-and-actual-provincial-expenditure

4. Davén J, Khoele A, Manamela L. 2020 HIV/AIDS Component of the HIV, TB, Malaria and Community Outreach Grant - an analysis of expenditure per output and options for future reform. Pretoria; 2021. Available: https://www.gtac.gov.za/pepa/wp-content/uploads/2021/11/HIV-TB-Spending-Review-Report.pdf

5. Barnabas R V, Szpiro AA, van Rooyen H, Asiimwe S, Pillay D, Ware NC, et al. Community-based antiretroviral therapy versus standard clinic-based services for HIV in South Africa and Uganda (DO ART): a randomised trial. Lancet Glob Heal. 2020;8: e1305–e1315. doi:10.1016/S2214-109X(20)30313-2

6. South Africa Department of Public Service and Administration. Appendices A to H4 to COLA 2021: 2019 and 2021 salary scales, with translation keys, for employees on salary levels 1 to 12 and those employees covered by Occupation Specific Dispensations (OSDs). In: South Africa Department of Public Service and Administration [Internet]. 2021 [cited 8 Feb 2024]. Available: https://www.dpsa.gov.za/dpsa2g/documents/rp/2021/Appendices A to H4 to COLA 2021.xlsx

7. South Africa National Department of Health. Current Master Health Product List – 1 January 2024. In: South Africa National Department of Health [Internet]. 2024 [cited 15 Jan 2024]. Available: https://www.health.gov.za/tenders/

8. U.S. National Library of Medicine. LABEL: ATRIPLA- efavirenz, emtricitabine, and tenofovir disoproxil fumarate tablet, film coated. In: National Library of Medicine [Internet]. 2021 [cited 15 Jan 2024]. Available: https://dailymed.nlm.nih.gov/dailymed/drugInfo.cfm?setid=2e97aa6d-09f7-46df-9499-63db7e9bac35

9. Meyer-Rath G, van Rensburg C, Chiu C, Leuner R, Jamieson L, Cohen S. The per-patient costs of HIV services in South Africa: Systematic review and application in the South African HIV Investment Case. McCreesh N, editor. PLoS One. 2019;14: e0210497. doi:10.1371/journal.pone.0210497

10. South African Department of Mineral Resources and Energy. Petrol Price Archive: Basic fuel price 2019. In: South African National Energy database [Internet]. 2020 [cited 15 Jan 2024]. Available: https://www.energy.gov.za/files/esources/petroleum/December2019/Basic-Fuel-Price.pdf

11. Bergg E. New Ford Ranger - Technical Specifications. 2016. Available: https://media.ford.com/content/dam/fordmedia/Europe/documents/productReleases/Ranger/FordRanger2016_TechnicalSpecifications_EU.pdf

12. The World Bank. GDP deflator (base year varies by country) - South Africa. In: The World Bank [Internet]. 2024 [cited 10 Dec 2023]. Available: https://data.worldbank.org/indicator/NY.GDP.DEFL.ZS?locations=ZA

13. Exchange Rates UK. US Dollar (USD) to South African Rand (ZAR) exchange rate history. In: Exchange Rates UK [Internet]. 2024 [cited 10 Dec 2023]. Available: https://www.exchangerates.org.uk/USD-ZAR-exchange-rate-history.html

14. U.S. Bureau of Labor Statistics. Databases, Tables & Calculators by Subject: Consumer Price Index for All Urban Consumers (CPI-U). In: U.S. Bureau of Labor Statistics [Internet]. 2024 [cited 19 Mar 2024]. Available: https://data.bls.gov/timeseries/CUUR0000SA0

15. UNAIDS. HIV sub-national estimates viewer. In: UNAIDS [Internet]. 2024 [cited 15 Feb 2024]. Available: https://naomi-spectrum.unaids.org/

16. UNAIDS. HIV Estimates with Uncertainty Bounds 1990-Present. 2023. Available: https://www.unaids.org/sites/default/files/media_asset/HIV_estimates_from_1990-to-present.xlsx

17. van Heerden A, Szpiro A, Ntinga X, Celum C, van Rooyen H, Essack Z, et al. A Sequential Multiple Assignment Randomized Trial of scalable interventions for ART delivery in South Africa: the SMART ART study. Trials. 2023;24: 32. doi:10.1186/s13063-022-07025-x

18. Guthrie T, Muheki C, Rosen S, Kanoowe S, Lagony S, Greener R, et al. Similar costs and outcomes for differentiated service delivery models for HIV treatment in Uganda. BMC Health Serv Res. 2022;22: 1315. doi:10.1186/s12913-022-08629-4

19. Prust ML, Banda CK, Nyirenda R, Chimbwandira F, Kalua T, Jahn A, et al. Multi‐month prescriptions, fast‐track refills, and community ART groups: results from a process evaluation in Malawi on using differentiated models of care to achieve national HIV treatment goals. J Int AIDS Soc. 2017;20. doi:10.7448/IAS.20.5.21650

20. Benade M, Nichols BE, Fatti G, Kuchukhidze S, Takarinda K, Mabhena-Ngorima N, et al. Economic evaluation of a cluster randomized, non-inferiority trial of differentiated service delivery models of HIV treatment in Zimbabwe. Pai M, editor. PLOS Glob Public Heal. 2023;3: e0000493. doi:10.1371/journal.pgph.0000493

21. Nichols BE, Cele R, Lekodeba N, Tukei B, Ngorima‐Mabhena N, Tiam A, et al. Economic evaluation of differentiated service delivery models for HIV treatment in Lesotho: costs to providers and patients. J Int AIDS Soc. 2021;24. doi:10.1002/jia2.25692
