# Supplementary material for: Costs of home-delivered antiretroviral therapy refills for persons living with HIV: Evidence from a pilot randomized controlled trial in KwaZulu-Natal, South Africa
Source: PLOS Glob Public Health. 2024 Dec 30;4(12):e0003368. doi: 10.1371/journal.pgph.0003368 (PMC11684705; doi:10.1371/journal.pgph.0003368)
Supplement: S3 File — (DOCX) [file pgph.0003368.s003.docx]

**S3 File: Additional results.**

Table of Contents

[3.1 Total annual cost of home-delivered ART under different scenarios 2](#_Toc167353495)

[3.2. Average annual per-client costs of home-delivered ART under different scenarios 3](#_Toc167353496)

[3.3. Total annual programmatic cost 4](#_Toc167353497)

[3.4. Cost drivers for the programmatic, 6-month refill scenario 5](#_Toc167353498)

[3.5. Cost drivers for the at-scale 3- and 6-month refill scenarios 8](#_Toc167353499)

# 3.1 Total annual cost of home-delivered ART under different scenarios

We estimated annual total programmatic cost based on South African National Department of Health (NDoH) costs and varied the salary sources and refill script durations. The total annual programmatic with 3-month refills was US $64,336 (**Supplementary Table 3.1.1**). In the 6-month refill script scenario with NDoH staff salaries, the total annual cost decreased to $49,538. In the 12-month refill script scenario with NDoH staff salaries, the total annual cost further decreased to $40,864. Scaling up the home delivery intervention increased total costs to $97,842 with 3-month refills, to $82,478 annually with 6-month refills, and to $87,843 annually with 12-month refills.

**Supplementary Table 3.1.1. Estimated total annual cost of home-delivered ART under different programmatic scenarios.**

| **Programmatic Scenario** | **Total annual cost** | |
| --- | --- | --- |
|  | 2022 ZAR | 2022 USD |
| Programmatic costs with 3-month refills^a^ | 1,053,454 | 64,336 |
| Programmatic costs with 6-month refills | 811,139 | 49,538 |
| Programmatic costs with 12-month refills | 669,115 | 40,864 |
| At-scale programmatic costs with 3-month refills^a^ | 1,602,080 | 97,842 |
| At-scale programmatic costs with 6-month refills | 1,350,519 | 82,478 |
| At-scale programmatic costs with 12-month refills | 1,438,355 | 87,843 |

^a^The standard-of-care in South Africa is 3-month ART refills at clinics.

# 3.2. Average annual per-client costs of home-delivered ART under different scenarios

The average annual Deliver Health Study costs per client and per client virally suppressed for quarterly refills in the first year of intervention are shown in **Supplementary Table 3.2.1**. Using staff salaries from the Deliver Health Study, the average annual cost per client and per client virally suppressed of home-delivered ART was $819 and $935, respectively, compared to $799 and $912, respectively, when substituting study expenses for NDoH costs.

**Supplementary Table 3.2.1. Estimated average annual costs of home-delivered ART under different implementation scenarios for quarterly refills in the first year of implementation.**

| **Scenario** | **Average annual cost per client** | | **Average annual cost per client virally suppressed** | |
| --- | --- | --- | --- | --- |
|  | 2022 ZAR | 2022 USD | 2022 ZAR | 2022 USD |
| With Deliver Health Study costs^a^ | 13,414 | 819 | 15,303 | 935 |
| With NDOH costs + staff salaries from the Deliver Health Study^a^ | 13,087 | 799 | 14,930 | 912 |

^a^The standard-of-care in South Africa is 3-month ART refills at clinics.

# 3.3. Total annual programmatic cost

The total annual cost of home delivery was $64,336 for the first year of intervention and $57,846 for subsequent years (**Supplementary Table 3.3.1**). In comparison, standard clinic-based ART refills and care was estimated to cost a total of $322,205,262 annually across KwaZulu-Natal.

**Supplementary Table 3.3.1. Total annual cost by ART refill method.**

| **Cost category** | **Total annual cost** | | | | | |
| --- | --- | --- | --- | --- | --- | --- |
|  | **Home delivery**^a^ | | | | **Clinic**^c,d^ | |
|  | First year^b^ | | Subsequent years | |  |  |
|  | 2022 ZAR | 2022 USD | 2022 ZAR | 2022 USD | 2022 ZAR | 2022 USD |
| ART drugs | 93,173 | 5,690 | 93,173 | 5,690 | 2,215,091,895 | 135,279,397 |
| Buildings and administrative overhead | 177,270 | 10,826 | 169,399 | 10,345 | 111,898,308 | 6,833,818 |
| Communication | 102,798 | 6,278 | 102,798 | 6,278 | 11,480,557 | 701,137 |
| Equipment | 97,189 | 5,936 | 12,181 | 744 | 3,402,951 | 207,824 |
| Hiring and training | 53,179 | 3,248 | 53,179 | 3,248 | 4,662,669 | 284,757 |
| Materials and supplies | 61,017 | 3,726 | 47,819 | 2,920 | 1,080,307,255 | 65,976,185 |
| Personnel wages and benefits | 352,713 | 21,541 | 352,713 | 21,541 | 1,848,506,990 | 112,891,438 |
| Vehicles and fuel | 120,799 | 7,377 | 120,609 | 7,366 | 502,772 | 30,705 |
| **Total** | 1,053,454 | 64,336 | 947,185 | 57,846 | 5,275,853,397 | 322,205,262 |

^a^There were 81 people living with HIV on ART in the home-delivered ART refills group of the Deliver Health Study.

^b^Includes startup costs.

^c^The standard-of-care in South Africa is 3-month ART refills at clinics.

^d^The total number of adults (aged 15 years and older) living with HIV in KwaZulu-Natal in September 2022 was 1,925,698 people. Source: [UNAIDS HIV sub-national estimates viewer](https://naomi-spectrum.unaids.org/).

# 3.4. Cost drivers for the programmatic, 6-month refill scenario

Increasing the ART refill scripts from 3- to 6-month refills, the ordering of top largest average annual costs per client and per client virally suppressed of home delivery were unchanged with personnel wages and benefits still the largest cost, followed by buildings and administrative overhead and vehicles and fuel in the first and subsequent years (**Supplementary Table 3.4.1**).

**Supplementary Table 3.4.1. Average annual cost per client and per client virally suppressed for home-delivered and clinic-based ART refills under the programmatic 6-month refills scenario.**

| **Cost category** | **Average annual cost per client** | | | | | | **Average annual cost per client virally suppressed** | | | | | |
| --- | --- | --- | --- | --- | --- | --- | --- | --- | --- | --- | --- | --- |
|  | **Home delivery**^a^ | | | | **Clinic**^c,d^  N=1,925,698 | | **Home delivery**^e^ | | | | **Clinic**^c,d^  N=1,270,961 | |
|  | First year^b^  N=81 | | Subsequent years  N=81 | |  |  | First year^b^  N=71 | | Subsequent years  N=71 | |  |  |
|  | 2022  ZAR | 2022  USD | 2022  ZAR | 2022  USD | 2022  ZAR | 2022  USD | 2022  ZAR | 2022  USD | 2022  ZAR | 2022  USD | 2022  ZAR | 2022  USD |
|  | Cost (% of total cost) | | | | | | | | | | | |
| ART drugs | 1,150 (11) | 70 (11) | 1,150 (12) | 70 (12) | 1,150 (42) | 70 (42) | 1,312 (18) | 80 (18) | 1,312 (20) | 80 (20) | 1,743 (42) | 106 (42) |
| Buildings and administrative overhead | 1,967 (20) | 120 (20) | 1,917 (21) | 117 (21) | 58 (2) | 4 (2) | 2,244 (30) | 137 (30) | 2,187 (33) | 134 (33) | 88 (2) | 5 (2) |
| Communication | 1,269 (13) | 78 (13) | 1,269 (14) | 78 (14) | 6 (0.22) | 0.36 (0.22) | 1,448 (20) | 88 (20) | 1,448 (22) | 88 (22) | 9 (0.22) | 0.55 (0.22) |
| Equipment | 1,200 (12) | 73 (12) | 150 (2) | 9 (2) | 2 (0.06) | 0.11 (0.06) | 1,369 (19) | 84 (19) | 172 (3) | 10 (3) | 3 (0.06) | 0.16 (0.06) |
| Hiring and training | 657 (7) | 40 (7) | 657 (7) | 40 (7) | 2 (0.09) | 0.15 (0.09) | 749 (10) | 46 (10) | 749 (11) | 46 (11) | 4 (0.09) | 0.22 (0.09) |
| Materials and supplies | 163 (2) | 10 (2) | 590 (6) | 36 (6) | 561 (20) | 34 (20) | 186 (3) | 11 (3) | 674 (10) | 41 (10) | 850 (20) | 52 (20) |
| Personnel wages and benefits | 2,177 (22) | 133 (22) | 2,177 (23) | 133 (23) | 960 (35) | 59 (35) | 2,484 (34) | 152 (34) | 2,484 (38) | 152 (38) | 1,454 (35) | 89 (35) |
| Vehicles and fuel | 1,489 (15) | 91 (15) | 1,484 (16) | 91 (16) | 0.26 (0.01) | 0.02 (0.01) | 1,699 (23) | 104 (23) | 1,693 (26) | 103 (26) | 0.40 (0.01) | 0.02 (0.01) |
| **Total**^f^ | 10,014 | 612 | 9,337 | 570 | 2,740 | 167 | 7,383 | 451 | 6,611 | 404 | 4,151 | 254 |

Abbreviations: ART = antiretroviral therapy, ZAR = South African Rand, USD = United States Dollars, HIV = Human Immunodeficiency Virus, UNAIDS = Joint United Nations Programme on HIV/AIDS.

^a^There were 81 people living with HIV on ART in the home-delivered ART refills group of the Deliver Health Study.

^b^Includes startup costs.

^c^The standard-of-care in South Africa is 3-month ART refills at clinics.

^d^The total number of adults (aged 15 years and older) living with HIV in KwaZulu-Natal in September 2022 was 1,925,698 people. Source: [UNAIDS HIV sub-national estimates viewer](https://naomi-spectrum.unaids.org/).

^e^There were 71 people living with HIV on ART who were virally suppressed at study exit in the home-delivered ART refills group of the Deliver Health Study.

^f^The average fee for home delivery service, about 4 USD, paid by clients in the home delivery intervention of the Deliver Health Study was subtracted from the programmatic costs of implementing home delivery.

(A)
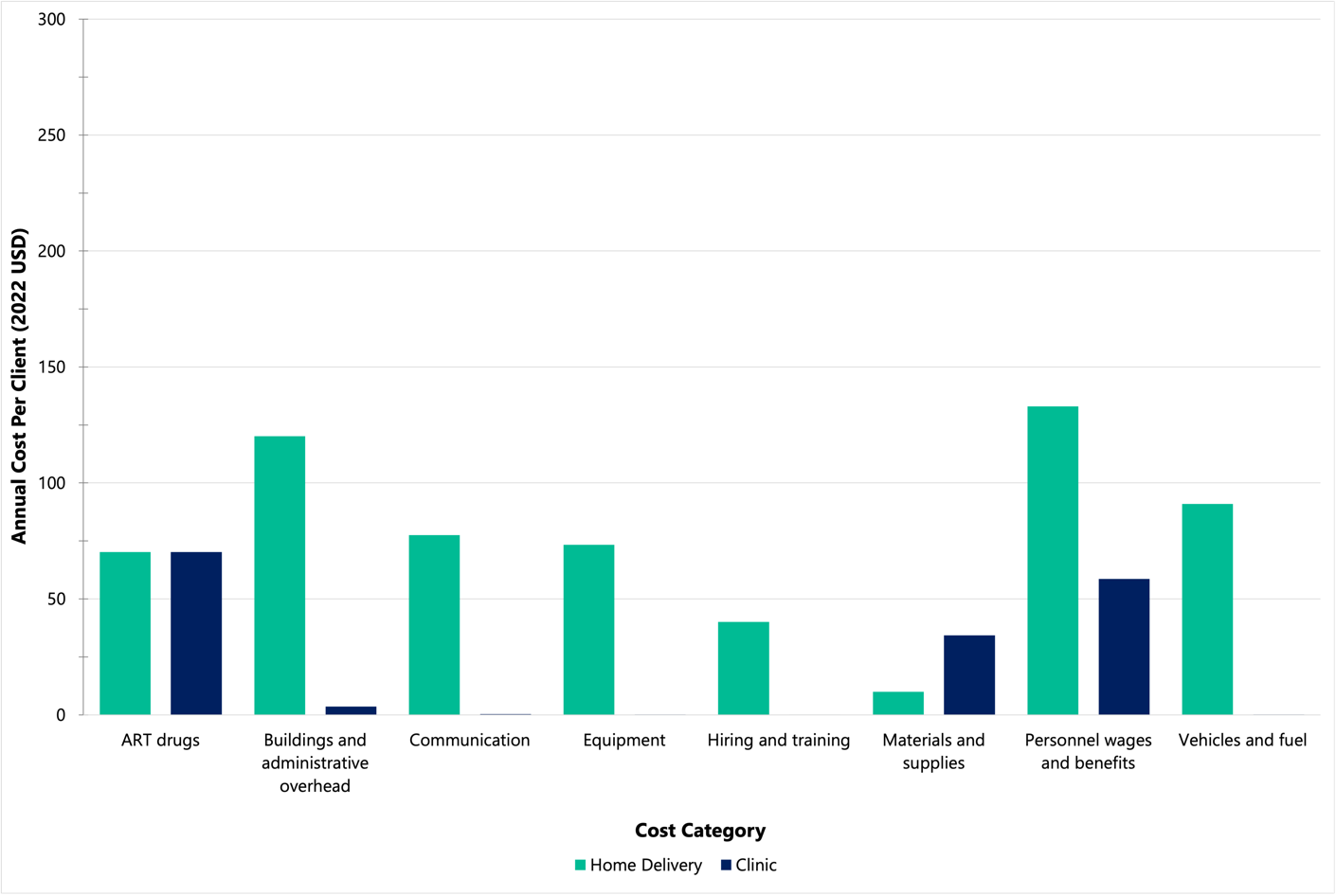


(B) **
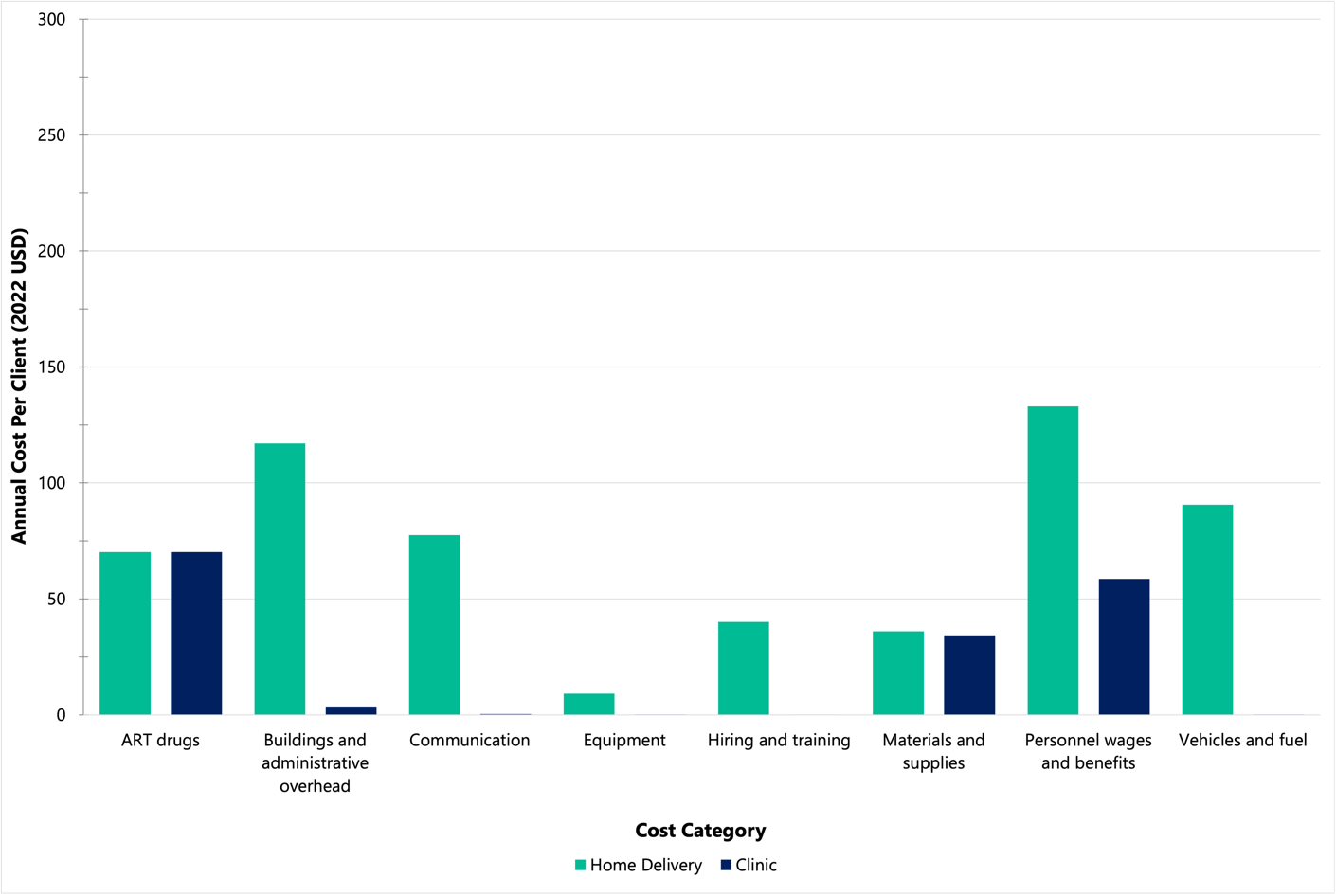
**

**Supplementary Figure 3.4.1. Average annual cost per client (2022 USD) for 6-month ART refills by cost category in the programmatic NDoH-implemented scenario.** The NDoH scenario assumes fixed costs as implemented in the Deliver Health Study and public sector clinical staff salaries instead of study salaries.

(A) Home-delivered ART intervention (first year costs) vs. clinic-based ART refills. (B) Home-delivered ART intervention (subsequent year costs) vs. clinic-based ART refills.

# 3.5. Cost drivers for the at-scale 3- and 6-month refill scenarios

When providing home-delivered 3-month ART refills at-scale, ART drugs became the greatest cost driver at 26%, different than the programmatic scenario (**Supplementary Table 3.5.1**). Personnel wages and benefits were the next largest cost of home delivery at 22%. Notice how the cost proportion of vehicles and fuel is reduced relative to other categories when home delivery is at-scale. The cost of all categories decreased when we increased the number of clients served by home-delivered ART refills.

**Supplementary Table 3.5.1. Average annual cost per client and per client virally suppressed for home-delivered and clinic-based ART refills under the at-scale 3-month refills scenario.**

| **Cost category** | **Average annual cost per client** | | | | | | **Average annual cost per client virally suppressed** | | | | | |
| --- | --- | --- | --- | --- | --- | --- | --- | --- | --- | --- | --- | --- |
|  | **Home delivery**^a^ | | | | **Clinic**^c,d^  N=1,925,698 | | **Home delivery**^e^ | | | | **Clinic**^c,d^  N=1,270,961 | |
|  | First year^b^  N=367 | | Subsequent years  N=642 | |  |  | First year^b^  N=323 | | Subsequent years  N=565 | |  |  |
|  | 2022  ZAR | 2022  USD | 2022  ZAR | 2022  USD | 2022  ZAR | 2022  USD | 2022  ZAR | 2022  USD | 2022  ZAR | 2022  USD | 2022  ZAR | 2022  USD |
|  | Cost (% of total cost) | | | | | | | | | | | |
| ART drugs | 1,150 (26) | 70 (26) | 1,150 (38) | 70 (38) | 1,150 (42) | 70 (42) | 1,307 (26) | 80 (26) | 1,307 (38) | 80 (38) | 1,743 (42) | 106 (42) |
| Buildings and administrative overhead | 531 (12) | 32 (12) | 306 (10) | 19 (10) | 58 (2) | 4 (2) | 603 (12) | 37 (12) | 347 (10) | 21 (10) | 88 (2) | 5 (2) |
| Communication | 280 (6) | 17 (6) | 160 (5) | 10 (5) | 6 (0.22) | 0.36 (0.22) | 318 (6) | 19 (6) | 182 (5) | 11 (5) | 9 (0.22) | 0.55 (0.22) |
| Equipment | 265 (6) | 16 (6) | 19 (0.63) | 1 (0.63) | 2 (0.06) | 0.11 (0.06) | 301 (6) | 18 (6) | 22 (0.63) | 1 (0.63) | 3 (0.06) | 0.16 (0.06) |
| Hiring and training | 145 (3) | 9 (3) | 83 (3) | 5 (3) | 2 (0.09) | 0.15 (0.09) | 165 (3) | 10 (3) | 94 (3) | 6 (3) | 4 (0.09) | 0.22 (0.09) |
| Materials and supplies | 753 (17) | 46 (17) | 590 (20) | 36 (20) | 561 (20) | 34 (20) | 856 (17) | 52 (17) | 671 (20) | 41 (20) | 850 (20) | 52 (20) |
| Personnel wages and benefits | 961 (22) | 59 (22) | 549 (18) | 34 (18) | 960 (35) | 59 (35) | 1,093 (22) | 67 (22) | 624 (18) | 38 (18) | 1,454 (35) | 89 (35) |
| Vehicles and fuel | 338 (8) | 21 (8) | 196 (7) | 12 (7) | 0.26 (0.01) | 0.02 (0.01) | 385 (8) | 23 (8) | 223 (7) | 14 (7) | 0.40 (0.01) | 0.02 (0.01) |
| **Total**^f^ | 4,367 | 267 | 2,996 | 183 | 2,740 | 167 | 4,963 | 303 | 3,405 | 208 | 4,151 | 254 |

Abbreviations: ART = antiretroviral therapy, ZAR = South African Rand, USD = United States Dollars, HIV = Human Immunodeficiency Virus, UNAIDS = Joint United Nations Programme on HIV/AIDS.

^a^There were 81 people living with HIV on ART in the home-delivered ART refills group of the Deliver Health Study.

^b^Includes startup costs.

^c^The standard-of-care in South Africa is 3-month ART refills at clinics.

^d^The total number of adults (aged 15 years and older) living with HIV in KwaZulu-Natal in September 2022 was 1,925,698 people. Source: [UNAIDS HIV sub-national estimates viewer](https://naomi-spectrum.unaids.org/).

^e^There were 71 people living with HIV on ART who were virally suppressed at study exit in the home-delivered ART refills group of the Deliver Health Study.

^f^The average fee for home delivery service, about 4 USD, paid by clients in the home delivery intervention of the Deliver Health Study was subtracted from the programmatic costs of implementing home delivery.

**Supplementary Table 3.5.2. Average annual cost per client and per client virally suppressed for home-delivered and clinic-based ART refills under the at-scale 6-month refills scenario.**

| **Cost category** | **Average annual cost per client** | | | | | | **Average annual cost per client virally suppressed** | | | | | |
| --- | --- | --- | --- | --- | --- | --- | --- | --- | --- | --- | --- | --- |
|  | **Home delivery**^a^ | | | | **Clinic**^c,d^  N=1,925,698 | | **Home delivery**^e^ | | | | **Clinic**^c,d^  N=1,270,961 | |
|  | First year^b^  N=367 | | Subsequent years  N=642 | |  |  | First year^b^  N=323 | | Subsequent years  N=565 | |  |  |
|  | 2022  ZAR | 2022  USD | 2022  ZAR | 2022  USD | 2022  ZAR | 2022  USD | 2022  ZAR | 2022  USD | 2022  ZAR | 2022  USD | 2022  ZAR | 2022  USD |
|  | Cost (% of total cost) | | | | | | | | | | | |
| ART drugs | 1,150 (31) | 70 (31) | 1,150 (40) | 70 (40) | 1,150 (42) | 70 (42) | 1,307 (31) | 80 (31) | 1,307 (40) | 80 (40) | 1,743 (42) | 106 (42) |
| Buildings and administrative overhead | 480 (13) | 29 (13) | 299 (10) | 18 (10) | 58 (2) | 4 (2) | 546 (13) | 33 (13) | 339 (10) | 21 (10) | 88 (2) | 5 (2) |
| Communication | 280 (8) | 17 (8) | 160 (6) | 10 (6) | 6 (0.22) | 0.36 (0.22) | 318 (8) | 19 (8) | 182 (6) | 11 (6) | 9 (0.22) | 0.55 (0.22) |
| Equipment | 265 (7) | 16 (7) | 19 (1) | 1 (1) | 2 (0.06) | 0.11 (0.06) | 301 (7) | 18 (7) | 22 (1) | 1 (1) | 3 (0.06) | 0.16 (0.06) |
| Hiring and training | 145 (4) | 9 (4) | 83 (3) | 5 (3) | 2 (0.09) | 0.15 (0.09) | 165 (4) | 10 (4) | 94 (3) | 6 (3) | 4 (0.09) | 0.22 (0.09) |
| Materials and supplies | 163 (4) | 10 (4) | 590 (20) | 36 (20) | 561 (20) | 34 (20) | 185 (4) | 11 (4) | 671 (20) | 41 (20) | 850 (20) | 52 (20) |
| Personnel wages and benefits | 919 (25) | 56 (25) | 465 (16) | 28 (16) | 960 (35) | 59 (35) | 1,045 (25) | 64 (25) | 528 (16) | 32 (16) | 1,454 (35) | 89 (35) |
| Vehicles and fuel | 336 (9) | 21 (9) | 191 (7) | 12 (7) | 0.26 (0.01) | 0.02 (0.01) | 382 (9) | 23 (9) | 217 (7) | 13 (7) | 0.40 (0.01) | 0.02 (0.01) |
| **Total**^f^ | 3,681 | 225 | 2,900 | 177 | 2,740 | 167 | 4,183 | 255 | 3,296 | 201 | 4,151 | 254 |

Abbreviations: ART = antiretroviral therapy, ZAR = South African Rand, USD = United States Dollars, HIV = Human Immunodeficiency Virus, UNAIDS = Joint United Nations Programme on HIV/AIDS.

^a^There were 81 people living with HIV on ART in the home-delivered ART refills group of the Deliver Health Study.

^b^Includes startup costs.

^c^The standard-of-care in South Africa is 3-month ART refills at clinics.

^d^The total number of adults (aged 15 years and older) living with HIV in KwaZulu-Natal in September 2022 was 1,925,698 people. Source: [UNAIDS HIV sub-national estimates viewer](https://naomi-spectrum.unaids.org/).

^e^There were 71 people living with HIV on ART who were virally suppressed at study exit in the home-delivered ART refills group of the Deliver Health Study.

^f^The average fee for home delivery service, about 4 USD, paid by clients in the home delivery intervention of the Deliver Health Study was subtracted from the programmatic costs of implementing home delivery.

(A)
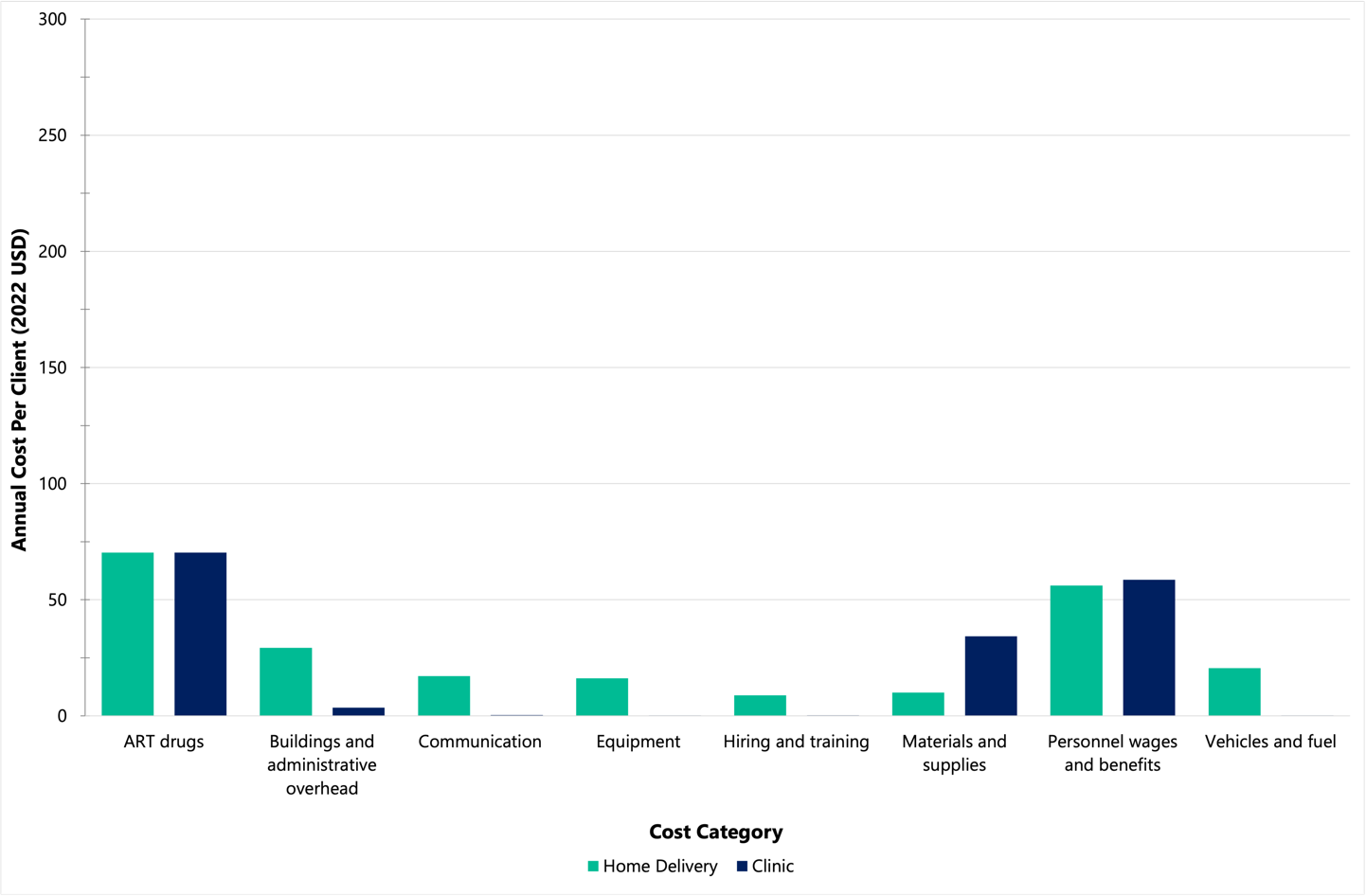


(B)
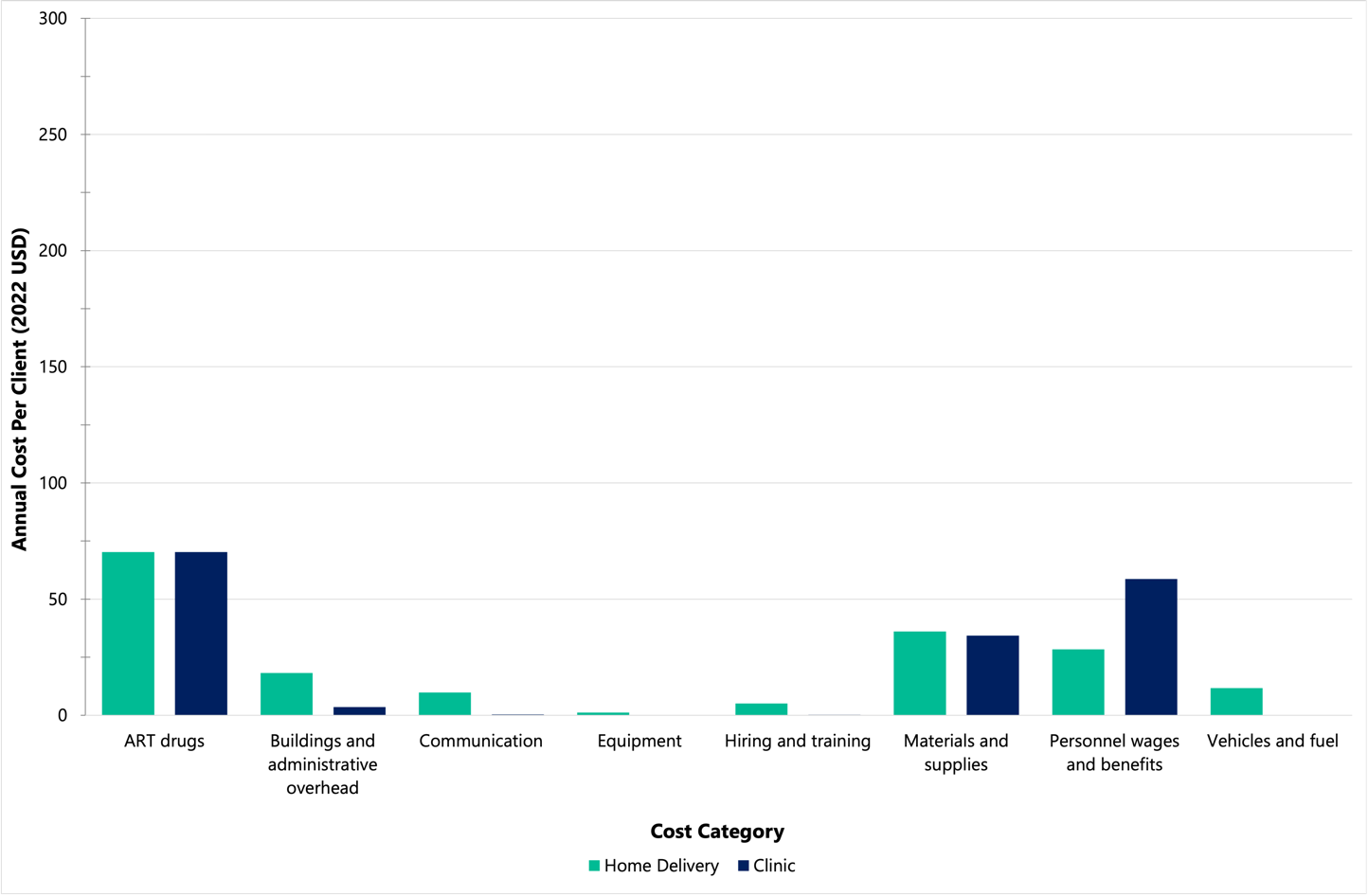


**Supplementary Figure 3.5.1. At-scale average annual cost per client (2022 USD) for 6-month ART refills by cost category in the programmatic NDoH-implemented scenario.** The NDoH scenario assumes fixed costs as implemented in the Deliver Health Study and public sector clinical staff salaries instead of study salaries. (A) Home-delivered ART intervention (first year costs) vs. clinic-based ART refills. (B) Home-delivered ART intervention (subsequent year costs) vs. clinic-based ART refills.
