# Supplementary material for: Costs of home-delivered antiretroviral therapy refills for persons living with HIV: Evidence from a pilot randomized controlled trial in KwaZulu-Natal, South Africa
Source: PLOS Glob Public Health. 2024 Dec 30;4(12):e0003368. doi: 10.1371/journal.pgph.0003368 (PMC11684705; doi:10.1371/journal.pgph.0003368)
Supplement: Attachment — Submitted filename: Reviewed papers- 2024.docx [file pgph.0003368.s004.docx]

**Title: Costs of home-delivered antiretroviral therapy refills for persons living with HIV: evidence from a pilot randomized controlled trial in KwaZulu-Natal, South Africa** Whereas home based services are good, they might be affected by stigma. They’re instances where a patient prefers to get medicines at distant places where they are not easily noticed by neighbours. Did the author think about this??

On 167 and 168, how did you cater for salaries of private facilities, it’s common for such facilities not to disclose their salary scales.

On 223, the sentence seems not well write- Consider revisiting it.

On 249, the justification for costing buildings under home based care is not clear. If we costed them, there is high possibility of variations in the cost since people have different buildings which would not be the case with health facilities were we expect standard structures. No wonder, buildings is the second largest driver in your analysis.

The proposed estimates possibly were hinged on assumption that patients do not shift, where they do shift, it’s likely the costs on transport incurred by a payer is likely to increase. We might need to indicate this under limitation of the study
