# Supplementary material for: Costs of home-delivered antiretroviral therapy refills for persons living with HIV: Evidence from a pilot randomized controlled trial in KwaZulu-Natal, South Africa
Source: PLOS Glob Public Health. 2024 Dec 30;4(12):e0003368. doi: 10.1371/journal.pgph.0003368 (PMC11684705; doi:10.1371/journal.pgph.0003368)
Supplement: Attachment — Submitted filename: Response to Reviewers_09-26-2024.docx [file pgph.0003368.s005.docx]

Response to Reviewer Comments

PLOS Global Public Health Manuscript Number: PGPH-D-24-01215

Title: Costs of home-delivered antiretroviral therapy refills for persons living with HIV: evidence from a pilot randomized controlled trial in KwaZulu-Natal, South Africa

| **Section** | **Reviewer Comment** | **Author Response** |
| --- | --- | --- |
| **Reviewer 1** | | |
| Overarching Feedback | The paper is well written especially highlighting the importance of home-delivered antiretroviral therapy refills in enhancing accessibility to HIV care in rural areas. I note however, the authors do not readily avail the data unless when requested through writing. | Thank you for your review of our manuscript and thoughtful comments. While a formal request will need to be submitted for access to de-identified Deliver Health Study data and/or costing spreadsheets, we have provided all our costing estimation data sources and the appendix includes detailed justifications and calculations for all assumptions made to assist with reproducibility. |
|  | Whereas home based services are good, they might be affected by stigma. They’re instances where a patient prefers to get medicines at distant places where they are not easily noticed by neighbours. Did the author think about this?? | You raise an important point; however, this topic is out of scope for our study, which evaluates costs and not effectiveness.  While out of scope, potential stigma surrounding home-delivered ART is something we have thought about. It is possible that there are clients who prefer to pick up their ART refills from distant locations from their homes for fear of stigma by their neighbors. We are not suggesting that home delivery should replace all other differentiated service delivery options for ART refills. Rather, we emphasize the importance of offering people living with HIV multiple options that best fits their needs and preferences (i.e., a patient-centered approach to HIV care), so that they feel empowered and experience fewer (physical) barriers to their HIV care. The parent study, the Deliver Health Study, had a qualitative component. While participants viewed home delivery as helping one avoid the HIV stigma associated with in-person clinic visits, indeed, the risk of stigma or violence due to inadvertent disclosure of HIV status was presented as a barrier to home delivery acceptance despite discreet packaging being used with the deliveries. Implementing discreet delivery methods in addition to discreet packaging was a suggested solution from that study: <https://doi.org/10.1186/s12913-023-10533-4>. |
| Methods | On 167 and 168, how did you cater for salaries of private facilities, it’s common for such facilities not to disclose their salary scales. | Since we were costing from the provider perspective (specifically from the South Africa National Department of Health), we only considered public clinics funded by the South Africa National Department of Health, which pays its staff based on the publicly published salary scale. We added a note in the Methods section on cost estimation that only public facilities were included: “All costs were estimated from the perspective of the South African NDoH (provider) and represented the costs of a program implemented in the public sector, to inform the South African NDoH’s financial planning and strategy” (lines 152-154). We also added this as a limitation in our Discussion section: “Additionally, since the provider was the South African NDoH, we did not consider the costs if the intervention had been implemented in the private sector” (lines 393-394). |
| Results | On 223, the sentence seems not well write- Consider revisiting it. | Thank you for your suggestion. We have revised this sentence for clarity: “We found that ‘as-observed’ costs for home-delivered ART in the Deliver Health Study were slightly higher than ‘as-implemented’ costs in the primary programmatic scenario due to hiring costs in the study ($819 per client and $935 per client virally suppressed for quarterly refills in the first year of intervention; see Supplementary Table 3.2.1. in S3 File)” (lines 230-233). |
|  | On 249, the justification for costing buildings under home based care is not clear. If we costed them, there is high possibility of variations in the cost since people have different buildings which would not be the case with health facilities were we expect standard structures. No wonder, buildings is the second largest driver in your analysis. | Thank you for pointing this out. We have added an explanation for why home-delivered ART cost estimates would include buildings and equipment: “Building rental costs for storage of ART stock and supplies, and equipment costs for ART monitoring for clients were gathered from the implementing institution, the Human Sciences Research Council” (lines 174-176). |
| Discussion | The proposed estimates possibly were hinged on assumption that patients do not shift, where they do shift, it’s likely the costs on transport incurred by a payer is likely to increase. We might need to indicate this under limitation of the study | This is a good point. We have added this to our limitations section in the Discussion: “Sixth, our estimates assume that clients do not relocate. If clients moved to a further destination but wanted to maintain the home delivery service, then transportation costs incurred by the intervention payer would likely increase” (lines 395-397). |
| **Reviewer 2** | | |
| Methods | The costing perspective for this study is the provider's perspective. Using the term "payers" perspective is erroneous because the payer could be the provider or the client/services consumer. | Thank you for pointing this out. We have updated it to be the ‘provider’ perspective instead of the ‘payer’ perspective throughout the manuscript (see lines 46, 153, 391, 393). |
|  | It is not well explained why the home-based care has costs such as buildings and equipment. These would be expected at health facility level only. A better explanation needs to come out clearly. | Thank you for pointing this out. We have added an explanation for why home-delivered ART cost estimates would include buildings and equipment: “Building rental costs for storage of ART stock and supplies, and equipment costs for ART monitoring for clients were gathered from the implementing institution, the Human Sciences Research Council” (lines 174-176). |
|  | What is the basis for assuming 12 deliveries per day at scale? | In the “At-scale cost estimation” sub-section of Methods, we explained how we arrived at the assumption of 12 home deliveries per day for the at-scale scenario. In the Deliver Health Study, the maximum number of deliveries made in a given day was 16 visits and the average time per visit was 26 minutes (including driving). Based on these numbers and assuming an 8-hour workday, we estimated that the same team could reasonably make 12 home deliveries a day, if they spent about 6 hours making home deliveries to clients and about 2 hours to load and unload the delivery vehicle at the central office.  We have revised this sub-section to make our justification clearer: “In the Deliver Health Study, the maximum number of deliveries made in a given day was 16 visits and the average time per visit was 26 minutes (including driving). With this information and assuming 214 workdays per year (excluding weekends, holidays, and other leave), five workdays per week, and eight-hour workdays, we estimated that the same team could reasonably make 12 home deliveries per day, spending approximately six hours of their workday making home deliveries to clients and about two hours to load and unload the delivery vehicle at the central office” (lines 210-216). |
|  | Why didn't the authors estimate the cost of ART delivery for DTG (and compare it with TDF/FTC/EFV? | In the Deliver Health Study, all participants (across both home delivery and clinic-based refills groups) were prescribed the TDF/FTC/EFV regimen. Since this reflects the true clinical practices at the time and since the intervention of interest is the method of ART delivery (home delivery vs facility-based delivery), we opted to keep the ART regimens consistent between the two arms in the costing study.  We have highlighted in the limitations that future studies should incorporate the change in guidelines: “Fourth, we estimated costs for TDF/FTC/EFV (the regimen prescribed for Deliver Health Study participants); however, as of 2023, South Africa recommends ART regimens containing DTG for greater adherence and viral suppression [17,44] [45,46] – future costing studies should note this change in clinical guidelines” (lines 387-390). |
| **Reviewer 3** | | |
| Overarching Feedback | This study is well-executed, with clear articulation of the objectives, methods, and results. The discussion section effectively interprets the evidence and thoughtfully addresses the study's limitations. The authors highlight that while the unit costs for the observed home-delivery program in the study were initially high, these costs can be significantly reduced through scale- up with a higher patient volume and by increasing the duration of ART refills. As a result, the costs become comparable to those of clinic-based ART delivery. The findings of this study provide valuable insights that will be highly useful for national strategy cost-projections and for researchers exploring the costs and cost-effectiveness of innovative ART delivery methods. | Thank you for your review of our manuscript and the thoughtful comments. |
| Introduction | Line 74- ‘successful’ treatment is ambiguous. Based on context, you’re likely referring to the cost of achieving viral suppression- so say that: “cost of achieving viral suppression is higher due to xxx”, but if you mean enrollment and retention to care –specify that. | Thank you for pointing this out. We have revised the sentence to state “Access to HIV care is further constrained by slowing government funding which has not matched the level of demand [5], particularly in rural areas where the cost of achieving HIV viral suppression is higher due to logistical barriers, supply chain considerations, and staffing challenges [6]” (lines 75-78). |
|  | Paragraph 1: You’ve effectively highlighted the greater prevalence and higher costs associated with HIV treatment in rural areas. To further reinforce your argument, can you include any statistics on ART coverage in rural vs. urban areas? Demonstrating lower ART coverage in rural areas would underscore inequities and thus help argue the need for home-based ART delivery in those settings. | While there is a lack of specific statistics on ART coverage in rural vs. urban South Africa, we have added some additional background in paragraph 1 to emphasize ART coverage disparities in rural vs. urban South Africa: “Patients attending clinics in rural South Africa have reported greater ART availability and affordability barriers than those in urban areas [3], and ART uptake in rural KwaZulu-Natal was strongly negatively associated with distance from the nearest primary healthcare facility [4]” (lines 72-75). |
| Methods | Considering that vehicle and fuel costs represented one of the largest cost categories- please specify in your methods what type of vehicle costs are included: Are these costs related to a single car, multiple motorcycles, recurring reimbursements for public transport, or a combination of these? Providing this detail would be useful for those looking to replicate the study. | We agree this level of detail is important for reproducibility of our study. We described our detailed assumptions about vehicle and fuel costs in Supplementary Table 2.2.1. (for home-delivered ART refills and monitoring) and Supplementary Table 2.2.2. (for clinic-based ART refills and care) of the S2 File (section 2.2. Costing categories and sources). We added additional information about how we calculated vehicle costs for the home delivery intervention. Please refer to “S2 File_withTC.docx” for the changes that were made to the file and to “S2 File.docx” for the clean version with no Track Changes. |
|  | Lines 142-151: please specify the time period for which costs were retrospectively collected for both study arms. | We have specified the time period for which costs were retrospectively collected. The time period was the same for both study arms. We also included the time period during which we collected the data/conducted this costing study: “Due to limited capacity for costing during the trial (which was conducted during the COVID-19 pandemic), costs were collected retrospectively in December 2023–March 2024 for the period of October 2019–December 2020, and the home-delivery and SOC costs relied on different data sources representing different regions of KwaZulu-Natal, described further below” (line 148-152). |
| Results | Not vital, but if you’d like you could add data labels to your figures, including the % of total costs per arm. | Thank you for your suggestion. Since the number and % of total cost of each cost category are already included in Table 1, we chose to keep the figures cleaner and simpler for the reader. |
| Discussion | Lines 323-324: Differences in costs with HIV investment case- of $290 vs. $167- are significantly different—and that warrants an explanation. Without an explanation, readers might be left questioning the validity/applicability of findings. You might consider geo/demographic differences, differences in methods, perhaps costs of certain inputs have changed, or scope (ie perhaps some other costs have been included/excluded). | We have added a potential explanation for the difference in the total annual cost per client in our study vs. the HIV investment case: “Second, our estimates for the SOC using KwaZulu-Natal’s provincial budget data ($167 per client annually) are substantially lower than the estimates from the South African HIV investment case, which estimated that the average cost (in 2022 USD) for providing ART per client annually across South Africa was $290 [37] – a potential explanation for our higher estimated cost of SOC compared with the HIV investment case is that our study represents KwaZulu-Natal province while the HIV investment case is a country-wide average. Our estimated incremental cost of home-delivered ART would be lower and appear even less expensive if our SOC estimate reflected a rural setting or used the South Africa HIV investment case – adding validity to our interpretation that the incremental cost is modest” (lines 357-366). |
|  | Lines 327-332: Confusing. The first part of the sentence states that costs in your study ($183) were lower than those observed in the DO ART Study ($287). However, the next sentence implies that home delivery should be more expensive than community-based delivery. This creates a contradiction: the data presented suggests that home delivery was actually less expensive, but the explanation given suggests the opposite should be true. You should keep the insights you have written, saying that you expected inverse results because fuel/time, but [provide possible reasons for lower costs]. | Thank you for catching this error – it in fact should have stated that costs in our study were lower (not higher) than those observed in the DO ART Study. We have corrected this sentence and added potential explanations for what we observed in terms of lower costs of home-delivered ART vs. community-based ART delivery: “Surprisingly, our estimated at-scale annual per-client costs of ART with quarterly refills were lower than those observed in the DO ART Study, a study of community-based ART conducted in the same region of rural South Africa ($183 in this study, compared with $287 in the DO ART Study for subsequent years after adjusting for inflation as shown in S2 File) [33] – this likely reflects the reasonably low vehicle and fuel costs required for home delivery of ART. The DO ART follow-on longitudinal modelling study found that community-based ART cost-effectively improved HIV clinical outcomes among men and accordingly prevented new infections and improved outcomes among women [38]. Since the Deliver Health Study found that home-based ART delivery also increased viral suppression overall and among men with lower observed costs than in DO ART [15], we might anticipate that home delivery is also cost-effective, though a comprehensive economic evaluation is needed” (lines 366-376). |
|  | Line 333: remove second ‘that’ | Thank you for catching the error. We have removed the second ‘that’ (line 378). |
|  | Your methods for estimating scale-up costs are well reasoned, and you justify the 12 home deliveries/day as a reasonable amount. The estimation is a good starting point, but it would be important to acknowledge in discussion that this assumption should be tested in practice. Specifically, further research is warranted to observe whether 12 a day is indeed feasible at scale, how costs change in scale up – particularly with regard to shifts in fixed and variable costs- and how actual observation would account for real-life in/efficiencies and therefore be more accurate. | Thank you for your suggestion. We have added an acknowledgement of this estimation needing to be tested in the discussion: “Future research is warranted to observe whether 12 home deliveries a day per team is indeed feasible at-scale, how costs change in scale-up (particularly with regard to shifts in fixed and variable costs), how actual observation of intervention implementation would account for real-life inefficiencies and therefore be more accurate, and to explore the costs and outcomes of various innovative approaches compared to traditional clinic-based care” (lines 421-425). |
